# Reviewers in 2024

**DOI:** 10.1098/rsbl.2025.0148

**Published:** 2025-05-07

**Authors:** Louise Heathwaite

**Affiliations:** ^1^ Lancaster University, Lancaster, UK

In one of my first duties as the new Editor-in-Chief of *Biology Letters*, I am delighted to carry on the tradition of thanking our reviewers and co-reviewers for their work throughout 2024. The scientific rigour of our content is paramount and cannot be achieved without your vital support.

 This article is made permanent and citable by its digital object identifier (DOI). We strongly encourage you to quote your contribution when applying for tenure and grants, or other forms of research assessment. Where you have provided it, we have included your ORCID iD to ensure that your contribution can be unambiguously assigned to you. Additionally, we also recognize our reviewers through services, such as Publons, which is integrated with *Biology Letters*.

The Editorial Board and I look forward to working with you and new generations of reviewers throughout 2025.

Abdala V

Achterberg EJM

Acosta-Avalos D

Adams J

Adomako M

https://orcid.org/0000-0001-9734-5408

Agrawal A

https://orcid.org/0000-0003-0095-1220

Aich U

https://orcid.org/0000-0003-2576-0922

Albo MJ

https://orcid.org/0000-0003-4332-7612

Albon S

Aleixo A

https://orcid.org/0000-0002-7816-9725

Alford L

Allan B

https://orcid.org/0000-0002-5991-9711

Álvarez-Manzaneda I

Alward B

https://orcid.org/0000-0002-1403-5277

Anderson WG

Andrews AH

Antunes D

Araújo Marreiro Varela S

https://orcid.org/0000-0002-9947-4024

Arbuckle K

https://orcid.org/0000-0002-9171-5874

Aron S

https://orcid.org/0000-0002-1674-8828

Auer (Thonhauser) K

https://orcid.org/0000-0002-6399-1388

Baca M

https://orcid.org/0000-0003-2174-3914

Baird E

https://orcid.org/0000-0003-3625-3897

Baker J

Balcarcel A

https://orcid.org/0000-0002-3826-834X

Bandini E

https://orcid.org/0000-0002-8293-9171

Barrett M

Barriuso J

Bath E

https://orcid.org/0000-0002-2988-8061

Baumeister R

Baxter-Gilbert J

Bayly N

https://orcid.org/0000-0001-9326-1936

Beauchamp G

https://orcid.org/0000-0002-3393-9562

Beaudet A

https://orcid.org/0000-0002-9363-5966

Beaulieu M

Beck S

https://orcid.org/0000-0001-6426-1603

Becker D

https://orcid.org/0000-0003-4315-8628

Begall S

https://orcid.org/0000-0001-9907-6387

Bensch H

https://orcid.org/0000-0002-8449-9843

Benson J

http://orcid.org/0000-0002-3993-4340

Benton M

https://orcid.org/0000-0002-4323-1824

Bernard E

https://orcid.org/0000-0002-2304-1978

Bhambore A

https://orcid.org/0009-0002-8593-3231

Bhamla S

https://orcid.org/0000-0002-9788-9920

Bhandawat V

https://orcid.org/0000-0002-2608-0403

Bhattacharjee S

Bicknell RDC

Bininda-Emonds ORP

Biondi L

https://orcid.org/0000-0002-7501-0268

Biro L

Bisch-Knaden S

https://orcid.org/0000-0002-3424-8376

Bize P

Blanco S

https://orcid.org/0000-0001-8766-697X

Blanco-Touriñán N

https://orcid.org/0000-0003-4610-6110

Bolek M

Bonadonna F

Bonier F

https://orcid.org/0000-0001-7245-0244

Booth D

Booth W

https://orcid.org/0000-0003-2355-0702

Bortot M

https://orcid.org/0000-0002-5815-2053

Botting J

Botías C

https://orcid.org/0000-0002-3891-9931

Bouyoucos I

https://orcid.org/0000-0002-4267-1043

Bowie R

Bowling DL

https://orcid.org/0000-0002-5303-5472

Bracken A

Brakefield P

Brandl H

Breed G

Brehm A

Brelsfoard C

Brian Rogers

Briggs D

https://orcid.org/0000-0003-0649-6417

Brigham R

Brink K

https://orcid.org/0000-0001-6630-1525

Brischigliaro M

Briscoe N

Brochu C

Brown C

Brown G

https://orcid.org/0000-0002-2924-9040

Brusatte S

Buckup P

Burdett H

https://orcid.org/0000-0002-3909-2235

Burdfield-Steel E

Burraco P

https://orcid.org/0000-0002-9007-2643

Burzyński A

Buss D

https://orcid.org/0000-0001-5362-240X

Butler P

Butler R

https://orcid.org/0000-0003-2136-7541

Buzatto B

https://orcid.org/0000-0002-2711-0336

Byrd-Craven J

Byrne M

https://orcid.org/0000-0002-8902-9808

Bythell J

https://orcid.org/0000-0003-2416-9786

Caldwell H

Caldwell M

https://orcid.org/0000-0002-2377-3925

Calvin Riiska

https://orcid.org/0009-0009-9511-8346

Camacho J

Camerlink I

https://orcid.org/0000-0002-3427-2210

Capellini I

Cardoso M

Cardozo-Ferreira GC

Careau V

https://orcid.org/0000-0002-2826-7837

Carlson B

Caro T

https://orcid.org/0000-0001-6804-8519

Carpentier F

Carr C

Carr D

Carranza S

Carretero M

Carrier D

Carter A

Carter C

Caruana F

https://orcid.org/0000-0002-4631-7543

Carvalho S

Casagrande S

https://orcid.org/0000-0002-4264-8062

Castiglione S

Castro LF

https://orcid.org/0000-0001-7697-386X

Caves E

https://orcid.org/0000-0003-3497-5925

Cavin L

Cerrito P

Chadwick F

Chaine A

Chapuisat M

https://orcid.org/0000-0001-7207-199X

Chen D

Chiao C-C

https://orcid.org/0000-0001-9506-0230

Chimienti M

Chiu C-I

https://orcid.org/0000-0002-1603-0736

Christa G

https://orcid.org/0000-0002-9454-7351

Christofides S

https://orcid.org/0000-0002-3806-3416

Citron S

Clark R

https://orcid.org/0000-0002-1100-1856

Clarke H

Clary D

Cocroft R

Codd J

https://orcid.org/0000-0003-0211-1786

Colombelli-Negrel D

https://orcid.org/0000-0002-9572-1120

Comas M

Condamine F

https://orcid.org/0000-0003-1673-9910

Constantino P

https://orcid.org/0000-0003-3397-7961

Cook C

https://orcid.org/0000-0003-3310-2161

Cooper N

https://orcid.org/0000-0002-4667-1542

Correia E

Cory J

https://orcid.org/0000-0002-4637-6439

Crespel A

https://orcid.org/0000-0002-6351-9008

Croijmans I

Cueva del Castillo R

https://orcid.org/0000-0002-6385-0729

Cullen T

https://orcid.org/0000-0002-4261-1323

Curry RA

Cutler GC

Czaczkes T

https://orcid.org/0000-0002-1350-4975

Córdoba Aguilar A

https://orcid.org/0000-0002-5978-1660

D'Andrea R

da Silva J

https://orcid.org/0000-0001-5631-5421

Dadda M

Dalerum F

Daniel Marsh

Darmaillacq A-S

Davenport ER

Dean C

Dean N

Deaner R

DeCasien A

https://orcid.org/0000-0002-6205-5408

Dececchi A

https://orcid.org/0000-0001-7972-3866

Delroisse J

https://orcid.org/0000-0002-9233-6470

Demartsev V

https://orcid.org/0000-0002-1456-9789

den Brave F

Denoël M

https://orcid.org/0000-0002-3586-8323

DeSilva J

https://orcid.org/0000-0001-7010-1155

Dettmer A

Dheilly N

Dijkstra P

https://orcid.org/0000-0001-5382-225X

Dillon M

Dion-Côté AM

Dixon MM

https://orcid.org/0000-0002-6537-2214

Diz A

Doherty, J-F

Dolby G

https://orcid.org/0000-0002-5923-0690

Dominic G

https://orcid.org/0000-0001-5118-9817

Douhard M

https://orcid.org/0000-0001-9422-7270

Drage H

https://orcid.org/0000-0002-0759-5970

Drake A

Drucker M

Drumheller S

Duarte R

https://orcid.org/0000-0001-7059-3129

Duchêne DA

https://orcid.org/0000-0002-5479-1974

Duffield G

https://orcid.org/0000-0003-1373-7805

Duffy M

https://orcid.org/0000-0002-8142-0802

Dugas M

Dugas MB

Dukas R

https://orcid.org/0000-0003-4533-8542

Duncan E

Durso A

Duthie B

Earley R

https://orcid.org/0000-0003-3046-3455

Eberhart-Hertel L

Elek Z

Elizabeth P

https://orcid.org/0000-0002-4398-9272

Elliot-Kerr S

Emerson B

Emery Thompson M

https://orcid.org/0000-0003-2451-6397

Emily B

Epstein J

Er-Fu Y

https://orcid.org/0000-0003-2385-6402

Erthal RP

Espinosa J

https://orcid.org/0000-0003-0780-2762

Esposito G

https://orcid.org/0000-0002-9442-0254

Evans L

Everaert G

Evers S

https://orcid.org/0000-0002-2393-5621

Fan P-F

https://orcid.org/0000-0003-4747-5727

Farji-Brener A

https://orcid.org/0000-0001-7251-3866

Farnole P

Fast K

Ferguson S

Ferreira MG

Ferreira R

https://orcid.org/0000-0001-7774-5252

Ferri S

Festa-Bianchet M

Ficetola F

Fiedler K

Fietz J

Fink P

Finotto L

Fisher J

Fisher J

Fisher S

https://orcid.org/0000-0002-3132-1996

Fleischer R

Foster J

https://orcid.org/0000-0002-4444-2375

Foth C

https://orcid.org/0000-0002-9410-4569

Francis C

https://orcid.org/0000-0003-2018-4954

Francisco M

https://orcid.org/0000-0003-2083-7812

Frank A

Frank T

https://orcid.org/0000-0002-9329-2414

Franklin A

https://orcid.org/0000-0002-8650-8344

Franklin C

https://orcid.org/0000-0003-1315-3797

Franks B

https://orcid.org/0000-0002-7558-6718

Fraser K

Frasnelli E

https://orcid.org/0000-0003-0493-0048

Freiler M

Friberg U

https://orcid.org/0000-0001-6112-9586

Fricke C

Friesen C

Frishkoff L

Funston G

https://orcid.org/0000-0003-3430-4398

Fusani L

Gaillard J-M

Galetto L

Galis F

Gangestad S

Garbuglio de Oliveira A

Garcia S

https://orcid.org/0009-0007-9076-2138

Gardner A

https://orcid.org/0000-0002-1304-3734

Gatto E

https://orcid.org/0000-0001-8784-2961

Gawecka K

Geiser F

https://orcid.org/0000-0001-7621-5049

George BB

https://orcid.org/0000-0003-1775-1647

George E

Girard-Buttoz C

https://orcid.org/0000-0003-1742-4400

Giroud S

Glazier D

https://orcid.org/0000-0001-7164-1823

Goater C

Godoy JA

Godoy P

https://orcid.org/0000-0003-4519-5094

GodóL

Goerlich V

https://orcid.org/0000-0002-8586-4435

Goerres C

Goodheart J

https://orcid.org/0000-0003-0754-5751

Gottlieb D

Goubault M

Gout R

Grace B

https://orcid.org/0000-0003-0090-6649

Graham A

https://orcid.org/0000-0002-6580-2755

Granatosky M

https://orcid.org/0000-0002-6465-5386

Greeff J

https://orcid.org/0000-0003-2951-6791

Grether G

https://orcid.org/0000-0003-3441-0537

Griffith S

https://orcid.org/0000-0001-7612-4999

Grim T

https://orcid.org/0000-0002-5775-6269

Grozinger C

https://orcid.org/0000-0001-5738-1201

Gruber B

Gruner D

https://orcid.org/0000-0002-3153-4297

Grunst A

Guillette L

https://orcid.org/0000-0002-8777-6543

Gunderson A

https://orcid.org/0000-0002-0120-4246

Hailer F

Halser C

Han F

https://orcid.org/0000-0003-3399-4008

Han J

https://orcid.org/0000-0002-5941-9899

Hanley D

Harborne A

Harper E

https://orcid.org/0000-0002-1092-3867

Harris S

Harrison J

https://orcid.org/0000-0001-5223-216X

Harrison J

https://orcid.org/0000-0001-7752-6471

Hart D

https://orcid.org/0000-0002-4592-558X

Hart S

Hautefeuille M

Hayes T

https://orcid.org/0009-0005-5967-2199

He Z

Hechinger R

https://orcid.org/0000-0002-6633-253X

Hecht E

Hedin M

Hegemann A

https://orcid.org/0000-0002-3309-9866

Heiko K

https://orcid.org/0000-0003-1060-9832

Helantera H

https://orcid.org/0000-0002-6468-5956

Hendricks M

Henneberg M

Hennig J

https://orcid.org/0000-0001-6450-6906

Henshaw J

https://orcid.org/0000-0001-7306-170X

Herniou E

Herrera-Ubaldo H

Hetem R

https://orcid.org/0000-0003-1953-3520

Higham J

https://orcid.org/0000-0002-1133-2030

Hill P

https://orcid.org/0000-0002-6190-6426

Hiller T

https://orcid.org/0000-0003-4044-9956

Hills T

Hinako A

https://orcid.org/0009-0000-9847-1393

Hinchcliffe J

Hines H

https://orcid.org/0000-0003-0299-5569

Hixon S

https://orcid.org/0000-0001-6147-7118

Hoelzl F

Holman L

https://orcid.org/0000-0002-7268-2173

Holmes J

https://orcid.org/0000-0001-8804-2149

Holtz T

Homma K

Hone D

https://orcid.org/0000-0002-5668-4603

Horn A

https://orcid.org/0000-0001-8850-6556

Horta-Lacueva Q

Houslay T

https://orcid.org/0000-0001-5592-9034

How M

https://orcid.org/0000-0001-5135-8828

Hozer C

https://orcid.org/0000-0001-8623-792X

Hu H

Hu Y

Huang K

https://orcid.org/0000-0002-0378-5988

Huey R

https://orcid.org/0000-0002-4962-8670

Huot P

Huynh J-R

Hörberg T

Immonen E

https://orcid.org/0000-0003-1121-6950

Inouye D

Iwaniuk A

https://orcid.org/0000-0001-9273-3655

Izquierdo-López A

https://orcid.org/0000-0002-8072-6308

Janecka M

Jarrett B

https://orcid.org/0000-0003-2071-6076

Jayaram K

Jean M

Jelbert S

Jennings D

https://orcid.org/0000-0001-5369-3574

Jessop A-L

https://orcid.org/0000-0001-8882-0027

Johansson C

https://orcid.org/0000-0002-1851-3635

John L

Johnson D

Johnson S

Johnston I

https://orcid.org/0000-0001-8559-3519

Jolly C

https://orcid.org/0000-0002-5234-0897

Jondelius U

Jordaan A

Jose M

https://orcid.org/0000-0001-8497-6681

Joseph F

https://orcid.org/0000-0002-7515-4632

Joseph L

https://orcid.org/0000-0001-7564-1978

Jude W

Juliana V

https://orcid.org/0000-0001-8357-4736

Jónsson Z

Kacelnik A

Kakui K

https://orcid.org/0000-0003-4630-9065

Kalinkat G

https://orcid.org/0000-0003-3529-5681

Kamenski P

Kammenga J

Kang C

https://orcid.org/0000-0003-3707-4989

Kappeler P

https://orcid.org/0000-0002-4801-487X

Kaufmann J

Kean D

Kear B

https://orcid.org/0000-0002-3128-3141

Keating R

Kelly A

https://orcid.org/0000-0002-6013-3943

Kelly C

https://orcid.org/0000-0002-0693-7211

Kemp D

https://orcid.org/0000-0002-5845-5513

Kempenaers B

Kepas M

Kevin C

Kingma SA

Kirschel A

https://orcid.org/0000-0003-4379-7956

Klug C

https://orcid.org/0000-0002-4099-7453

Kobayashi K

Koblmueller S

Koch E

Kohlmeier P

https://orcid.org/0000-0002-7123-9209

Konarzewski M

https://orcid.org/0000-0001-7428-6521

Konczal M

Koppik M

Krabbenhoft T

Kramer J

https://orcid.org/0000-0003-2155-9295

Krasheninnikova A

https://orcid.org/0000-0001-8566-8277

Kronauer D

https://orcid.org/0000-0002-4103-7729

Kurtz J

https://orcid.org/0000-0002-7258-459X

Kučera M

Kyle D

Kynan D

Körner M

https://orcid.org/0000-0001-9086-4731

Ladwa S

Lai S

https://orcid.org/0000-0003-0128-3738

Laidre K

http://orcid.org/0000-0002-2787-650X

Lameira A

https://orcid.org/0000-0003-3071-4824

Lampei C

Lamsdell J

https://orcid.org/0000-0002-1045-9574

Lang B

Laskowski K

https://orcid.org/0000-0003-1523-9340

Latch E

Laumer C

Laurie J

Lavretsky P

https://orcid.org/0000-0002-5904-8821

Le Gall M

https://orcid.org/0000-0002-8358-0332

Leahy B

Leberg P

LeBoeuf A

https://orcid.org/0000-0002-2931-1510

Lehtonen J

https://orcid.org/0000-0001-5260-1041

Leips J

Lesser M

Li G

https://orcid.org/0000-0002-4423-3266

Liao J

https://orcid.org/0000-0002-9520-3235

Libbrecht R

https://orcid.org/0000-0003-4397-000X

Liebal K

https://orcid.org/0000-0003-2447-8327

Lienard M

Lima C

https://orcid.org/0000-0003-3058-7204

Lima M

https://orcid.org/0000-0002-8952-3080

Linacre A

Llaurens V

Lo Parrino E

Locatello L

https://orcid.org/0000-0002-3530-2565

Lochlan W

https://orcid.org/0000-0002-3771-474X

Lockery S

https://orcid.org/0000-0001-8535-7989

Lockyer C

Lohmann K

Long T

https://orcid.org/0000-0002-8708-2728

Longo A

https://orcid.org/0000-0002-5112-1246

Longrich N

https://orcid.org/0000-0002-9586-8907

Loosli F

Lopez-Uribe MM

https://orcid.org/0000-0002-8185-2904

Loudon A

Loukola O

https://orcid.org/0000-0002-9094-2004

Lukes J

Lunau K

https://orcid.org/0000-0001-5184-4201

Lyamin O

https://orcid.org/0000-0003-2049-0943

Lymbery S

https://orcid.org/0000-0002-8801-7880

Lynch K

Lüpold S

https://orcid.org/0000-0002-5069-1992

Machanda Z

https://orcid.org/0000-0001-7060-7949

Macías García C

https://orcid.org/0000-0003-3242-4214

Maeda T

https://orcid.org/0000-0002-1066-7314

Mainwaring MC

Malerba ME

https://orcid.org/0000-0002-7480-4779

Maloney SK

https://orcid.org/0000-0002-5878-2266

Manafzadeh A

https://orcid.org/0000-0001-5388-7942

Marcantonio M

https://orcid.org/0000-0003-3896-2355

Marcos-Torres FJ

Marek L

https://orcid.org/0000-0001-6370-8880

Mariette M

https://orcid.org/0000-0003-0567-4111

Markotter W

Marques T

Marques V

Martin-Ordas G

https://orcid.org/0000-0002-5221-9181

Martinossi-allibert I

https://orcid.org/0000-0002-8332-8620

Martins G

https://orcid.org/0000-0003-0614-8551

Martyka R

https://orcid.org/0000-0002-0676-6432

Martín H

Martín-Maldonado B

Mashanov V

Mason M

Matisoo-Smith L

Matsuzawa T

https://orcid.org/0000-0002-8147-2725

Matthiopoulos J

https://orcid.org/0000-0003-3639-8172

May S

Maziarz M

https://orcid.org/0000-0002-2921-5713

Mazzoni V

https://orcid.org/0000-0003-4036-5322

McClelland G

McCoy K

McCullough E

McDaniel S

https://orcid.org/0000-0002-5435-7377

McGee M

McGettigan C

https://orcid.org/0000-0001-6293-3795

McGowan C

McGrosky A

McMahon K

McMichael L

Meade J

https://orcid.org/0000-0003-1082-9907

Measey GJ

Medina I

https://orcid.org/0000-0002-1021-5035

Mella V

https://orcid.org/0000-0001-5577-2487

Mendl M

https://orcid.org/0000-0002-5302-1871

Michaud M

https://orcid.org/0000-0001-7075-9862

Miguel MA

https://orcid.org/0000-0003-4369-2898

Miller P

https://orcid.org/0000-0001-8028-9673

Miller Z

Milutinović B

Miranda R

Mizusawa K

Molento C

Moleón M

https://orcid.org/0000-0002-3126-619X

Mollon J

https://orcid.org/0000-0001-8533-033X

Momo F

https://orcid.org/0000-0003-0710-1149

Mongiardino Koch N

https://orcid.org/0000-0001-6317-5869

Montgomerie R

https://orcid.org/0000-0003-4701-4525

Montgomery A

https://orcid.org/0000-0002-3430-3451

Moore B

Moraes AP

Morales-Reyes Z

https://orcid.org/0000-0002-4529-8651

Moret Y

https://orcid.org/0000-0002-2435-8416

Morris M

https://orcid.org/0000-0001-9962-2982

Morrison R

https://orcid.org/0000-0001-9161-4734

Motta E

https://orcid.org/0000-0001-9360-4353

Muhlfeld C

Munakata

https://orcid.org/0009-0009-4458-7281

Muposhi V

Murali G

https://orcid.org/0000-0002-5307-325X

Murphy P

https://orcid.org/0000-0003-1321-8749

Müller J

Müller R

https://orcid.org/0000-0001-8894-9875

Nadeau N

https://orcid.org/0000-0002-9319-921X

Naggar YA

Nandy B

https://orcid.org/0000-0002-9588-0316

Nanglu K

Naoya A

Narayan E

Narendra A

https://orcid.org/0000-0002-1286-5373

Nascimento Corte G

Nash C

Navarro N

Neate-Clegg M

https://orcid.org/0000-0001-9753-6765

Neves F

Newell FL

https://orcid.org/0000-0002-7944-8603

Nichols H

https://orcid.org/0000-0002-4455-6065

Nishikawa Y

Nitta J

Nityananda V

https://orcid.org/0000-0002-2878-2425

Nneji L

Nourani E

https://orcid.org/0000-0003-4420-3902

Nufio C

Nunn C

https://orcid.org/0000-0001-9330-2873

Oakley CA

Oakley TH

https://orcid.org/0000-0002-4478-915X

Ocampo D

https://orcid.org/0000-0003-1597-4038

Oda G

https://orcid.org/0000-0001-7776-5368

Oesterle J

Ogawa Y

Ohmer M

https://orcid.org/0000-0002-5937-6585

Okada Y

https://orcid.org/0000-0003-4528-2980

Opazo, JC

https://orcid.org/0000-0001-7938-4083

Organ C

https://orcid.org/0000-0002-2814-2217

Orlova M

Ormerod S

Ørsted M

https://orcid.org/0000-0001-8222-8399

Orzack S

Osiejuk T

Owens R

Owerkowicz T

Pageat P

Palagi E

https://orcid.org/0000-0002-2038-4596

Pamenter M

https://orcid.org/0000-0003-4035-9555

Passamonti M

Passos DC

Patlar B

https://orcid.org/0000-0002-0442-9061

Pattrick J

https://orcid.org/0000-0001-6587-5500

Pavón-Vázquez C

Peckre L

Pekar S

https://orcid.org/0000-0002-0197-5040

Pelletier D

Pellis S

Penalba J

https://orcid.org/0000-0001-6549-6885

Peng F

https://orcid.org/0000-0002-1637-5611

Pepperberg I

https://orcid.org/0000-0001-9486-7323

Perez Castello P

Perkin E

Perlut N

Perrot-Minnot M-J

https://orcid.org/0000-0003-3412-4282

Peter C

Petsios E

https://orcid.org/0000-0002-7955-7412

Pilakouta N

https://orcid.org/0000-0001-8503-520X

Pincebourde S

Pineda-Pampliega J

https://orcid.org/0000-0003-4997-5098

Pinto-Zevallos D

Pipoly I

https://orcid.org/0000-0002-4541-6056

Pirk C

Pizo M

Pomberger T

Ponti R

https://orcid.org/0000-0003-3485-5528

Potapov A

Powell D

https://orcid.org/0000-0001-9204-645X

Pozo Romero MI

Prakash A

Prasad G

Preston D

Pretto Flávio

Pruett J

Pulido Mantas T

Pusey A

Putman N

https://orcid.org/0000-0001-8485-7455

Puts D

Pérez Mellado V

Pérez-Cembranos A

https://orcid.org/0000-0002-8296-2283

Pérez-García A

Quakenbush L

Quintanilla M

Rabosky D

https://orcid.org/0000-0002-7499-8251

Racey P

Ramenofsky M

Ramos J

Rands S

https://orcid.org/0000-0002-7400-005X

Raphaëlle H

https://orcid.org/0009-0000-1422-2016

Rauschkolb R

Ravindran S

https://orcid.org/0000-0003-0996-0262

Rawlence N

https://orcid.org/0000-0001-6968-6643

Ray A

https://orcid.org/0000-0002-9354-4247

Ray Smith A

https://orcid.org/0000-0001-9951-7859

Rebelo R

Reboreda J

Reino L

https://orcid.org/0000-0002-9768-1097

Remacha C

Remeš V

Ren Z-X

https://orcid.org/0000-0001-7265-065X

Renaud L-A

https://orcid.org/0000-0001-8496-3979

Rey A

Reyes Prado H

Ridley A

https://orcid.org/0000-0001-5886-0992

Riedel J

https://orcid.org/0000-0002-3947-3679

Riley Wood

Rios-Chelen A

Risueno-Segovia C

Rittschof D

Robillard T

https://orcid.org/0000-0002-2177-9549

Rogers J

Rogers L

https://orcid.org/0000-0002-9956-1769

Rokyta D

Rolff J

https://orcid.org/0000-0002-1529-5409

Romero-Haro A

https://orcid.org/0000-0002-7127-4733

Ronget V

Rosati A

https://orcid.org/0000-0002-6906-7807

Rose, P

Rowland H

https://orcid.org/0000-0002-1040-555X

Royle N

https://orcid.org/0000-0002-1617-3884

Rozaimi M

https://orcid.org/0000-0001-6631-8677

Rutschmann B

https://orcid.org/0000-0001-6589-6408

Sakaguchi TF

Sakaluk S

Sakiyama T

https://orcid.org/0000-0002-2687-7228

Salin K

https://orcid.org/0000-0002-3368-9639

Sampaio E

https://orcid.org/0000-0002-9222-2553

Sanders D

https://orcid.org/0000-0003-2383-8693

Sappal R

Saranathan V

https://orcid.org/0000-0003-4058-5093

Sasaki M

https://orcid.org/0000-0001-5560-5363

Sato T

https://orcid.org/0000-0002-4660-9669

Sbragaglia V

https://orcid.org/0000-0002-4775-7049

Schäfer R

Scharf H

https://orcid.org/0000-0002-4385-3850

Scharf I

https://orcid.org/0000-0002-8506-7161

Schausberger P

https://orcid.org/0000-0002-1529-3198

Scherb H

Scheuerlein A

Schilthuizen M

Schlosser G

Schlüter P

Schmachtenberg O

Schmidt-Lebuhn A

http://orcid.org/0000-0002-7402-8941

Schoepf V

Schott O

Schröder-Turk G

https://orcid.org/0000-0001-5093-415X

Schulte P

https://orcid.org/0000-0002-5237-8836

Schultner E

https://orcid.org/0000-0002-5069-9732

Schupp E

Schurch N

Sciscio L

Scofield R

https://orcid.org/0000-0002-7510-6980

Scott Persons 4th, W

Sebastianelli M

https://orcid.org/0000-0002-0858-8887

Seddaiu P

Sefc K

https://orcid.org/0000-0001-8108-8339

Serrano E

Seymour R

https://orcid.org/0000-0002-3395-0059

Seymoure B

https://orcid.org/0000-0003-3596-1832

Shafir S

https://orcid.org/0000-0002-9068-1078

Shahandeh M

Shanmugam S

Shawkey M

https://orcid.org/0000-0002-5131-8209

Shikanov A

Shimoji H

Shiojiri N

Short S

https://orcid.org/0000-0002-1074-8982

Shu L

https://orcid.org/0000-0001-9683-906X

Sibert E

https://orcid.org/0000-0003-0577-864X

Sigsgaard E

Simmons N

https://orcid.org/0000-0001-8807-7499

Slavík O

https://orcid.org/0000-0003-3443-4125

Sloman K

Slominski A

Smart U

Smith B

Smith H

Smith M

https://orcid.org/0000-0001-5660-1727

Smith T

https://orcid.org/0000-0002-5256-6087

Smithers S

Sneddon L

https://orcid.org/0000-0001-9787-3948

Snyder A

https://orcid.org/0009-0000-4922-2702

Soares B

Somanathan H

https://orcid.org/0000-0002-4803-3894

Sondhi Y

https://orcid.org/0000-0002-7704-3944

Speers-Roesch B

https://orcid.org/0000-0001-6510-7501

Sperling AL

https://orcid.org/0000-0001-6298-4832

Špinka M

Staes N

Star B

https://orcid.org/0000-0003-0235-9810

Steadman D

Stephanie Woodgate

Stewart-Williams S

Strang C

Strauss S

Stroud J

https://orcid.org/0000-0003-0734-6795

Stynoski J

https://orcid.org/0000-0002-9132-8567

Stärk J

Stöckl A

Sudeep R

Sudyka J

https://orcid.org/0000-0003-3006-3806

Sueur J

Sun S

https://orcid.org/0000-0003-0748-8048

Szekely T

https://orcid.org/0000-0003-2093-0056

Szep T

Taff C

https://orcid.org/0000-0003-1497-7990

Takeshita R

Talamoni SA

Tammaru T

https://orcid.org/0000-0002-6892-5910

Taverna E

Teets N

https://orcid.org/0000-0003-0963-7457

Temizyürek T

Thompson F

https://orcid.org/0000-0001-7581-2204

Thompson González N

https://orcid.org/0000-0002-3195-1277

Thorne L

Tietje M

https://orcid.org/0000-0003-1157-2963

Tobler M

https://orcid.org/0000-0001-5895-6302

Tom Foster

Tomášek O

https://orcid.org/0000-0002-2657-5916

Toomey M

https://orcid.org/0000-0001-9184-197X

Totten R

Townsend A

https://orcid.org/0000-0002-2452-1594

Traniello J

Treanore E

Tryjanowski P

https://orcid.org/0000-0002-8358-0797

Tsai C-H

https://orcid.org/0000-0003-3617-366X

Tuliozi B

https://orcid.org/0000-0001-5656-4858

Tungadi T

Tuya F

https://orcid.org/0000-0001-8316-5887

Tyers A

Tylzanowski P

Ugolini A

Uthicke S

Uwe M

Valenciano A

Van de Schoot E

https://orcid.org/0009-0004-0134-0952

Van Dyck H

Van Houcke J

van Toor M

https://orcid.org/0000-0002-2254-5779

Vanbergen A

https://orcid.org/0000-0001-8320-5535

Vannier J

https://orcid.org/0000-0003-0998-1231

Vasudeva R

https://orcid.org/0000-0002-3831-0384

Vaziri G

Veeraragavan S

Velando A

https://orcid.org/0000-0001-8909-0724

Veller C

Verhulst S

https://orcid.org/0000-0002-1143-6868

Vermeij G

https://orcid.org/0000-0002-6450-5687

Vigilant L

https://orcid.org/0000-0003-4509-1260

Vilcinskas A

https://orcid.org/0000-0001-8276-4968

Vilà C

Vincze O

Vinkler M

https://orcid.org/0000-0003-3572-9494

Vinther J

https://orcid.org/0000-0002-3584-9616

Vittecoq M

Vogt K

https://orcid.org/0000-0002-8763-342X

Vonk J

https://orcid.org/0000-0002-4362-5989

Votier SC

Vágási C

https://orcid.org/0000-0002-8736-2391

Wainwright DK

Waldman B

https://orcid.org/0000-0003-0006-5333

Walsh S

Waner Mariquito R

Wang Z

https://orcid.org/0000-0002-5734-7385

Ward D

Ward SJ

https://orcid.org/0000-0002-5857-1071

Watanabe A

https://orcid.org/0000-0001-5057-4772

Watanabe K

Watkins A

Watson D

Waybright S

https://orcid.org/0000-0003-0290-2772

Weinstein S

Weis V

Welch K

https://orcid.org/0000-0002-3283-6510

Werdelin L

Westerdahl H

https://orcid.org/0000-0001-7167-9805

Wheeler S

Whittaker B

https://orcid.org/0000-0001-9316-6285

Wiberg RAW

https://orcid.org/0000-0002-8074-8670

Wieder K

Wilkins A

Williams E

Williams K

https://orcid.org/0000-0003-2862-3974

Wilts B

https://orcid.org/0000-0002-2727-7128

Winkler L

https://orcid.org/0000-0003-3597-6540

Wisnoski N

https://orcid.org/0000-0002-2929-5231

Wojciechowski M

Wojczulanis-Jakubas K

Wolf B

https://orcid.org/0000-0001-5643-7867

Wolf F

https://orcid.org/0000-0002-0955-8487

Wolff J

https://orcid.org/0000-0003-2326-0326

Wooster E

https://orcid.org/0000-0002-2907-4076

Xi N

Xing L-X

Xu M

Xu S

https://orcid.org/0000-0002-1304-9968

Yamaguchi Y

https://orcid.org/0000-0001-7340-4557

Yamamichi M

https://orcid.org/0000-0003-2136-3399

Yan, C

https://orcid.org/0000-0002-6669-432X

Yang Y

Yao Y-G

Yin C

York J

https://orcid.org/0000-0003-4947-0591

Young J

https://orcid.org/0000-0001-5358-9339

Young M

https://orcid.org/0000-0002-7263-6505

Young R

Yu C

Zakaria M

https://orcid.org/0000-0003-2456-6415

Zarbiv Benita M

Zhou Y

https://orcid.org/0000-0003-3324-9972

Zimmermann H

https://orcid.org/0000-0002-7919-4447

Zlatar D

